# The Chichester Infirmary

**Published:** 1906-12-08

**Authors:** Norcliffe Gilpin

**Affiliations:** Chairman, Committee of Management. Chichester Infirmary


### Editor's Letter-Box.

### THE CHICHESTER INFIRMARY.

*To the Editor of* The Hospital.

Sir,—The attention of the Committee of Management of the Chichester Infirmary has been called to your notice of Colonel Garnham's attack upon them. As, whenever the sense of a general meeting of the Governors has been adverse to Colonel Garnham, he invariably sends a triplicate letter to the three local journals setting forth his grievances, it has become customary for the committee individually and collectively never to reply. But now that the attention of so important an organ in the charitable world as The Hospital has been attracted by his last communication, I have been desired by the committee to state to you shortly the true facts of the case which, as set forth by the Colonel, is distinctly misleading. In consequence of an instruction to the committee passed at the half-yearly meeting of the Governors last April to prepare a new edition of the statutes, omitting obsolete rules and incorporating alterations already sanctioned, and generally bringing them into accord with existing practices—which was seconded by Colonel Garnham—such a revised edition of the statutes was prepared by the committee and brought before a *special meeting of the Governors* held on September 24th, when they were adopted after a full debate. At the annual meeting in November in accordance with the statute they were again brought up for confirmation and were duly confirmed. On both occasions Colonel Garnham was defeated in all his amendments except one small one, which was accepted by the committee. To most of them he could not get a seconder.

The attack upon the honorary staff is most ungenerous and misleading. The old Statute 35 had been obsolete for certainly more than thirty years, and its retention unaltered was objected to by the honorary medical officers. As accidents and urgent cases are admitted on all days and at all hours, nearly half the patients are not affected by the statute at all, and of those less urgent cases who enter on the Tuesday, the greater part have already been seen by one of the honorary staff or by some medical man in the neighbourhood, who has communicated with the house surgeon. The latter sees the patients and satisfies himself as to their medical fitness for admission and passes them on to the committee, who decide as to their fitness to become objects of the charity. Later in the day, or on the next morning, they are seen by the physician or the surgeon of the week, and the cases fully examined. This has been the practice for many years, and the committee have not felt it their duty to interfere, and perhaps to quarrel, with the honorary staff. With regard to the number of the latter, from early times the rule has been to have two physicians and two surgeons on the active list, *as is the case now*. Occasionally, under special circumstances, there have been three on one side or the other, but even then two out of the three have been sharing the work of one man. It is by statute left to the honorary staff to advise the committee as to the number required to do the work, and any representation on their part would receive immediate attention.

As regards the date of the annual meeting, the committee advised the return to the old date as being more convenient in many ways. Unfortunately on the last occasion the annual report was not ready to send out with the notice of meeting, entirely through the fault of the printer, who had had the corrected proofs in his hands in ample time. There was no difficulty about it, and it was a pure accident, not likely to happen again. With respect to the objection to the ruling of the Chairman at the special meeting, it is not the business of the committee to interfere with or to question the proceedings or the decisions of a general meeting of the Governors. The honorary solicitors were not asked about the point, and no instruction was given them to obtain counsel's opinion. The senior partner of the firm was present at the special meeting as a Governor, and not officially. *After* the Chairman had given his ruling he was allowed by the Chairman's courtesy—though quite out of order—to state his views. Other Governors belonging to the legal profession differ from him as to the interpretation of the statute, particularly as the same interpretation had been accepted without question on a previous occasion.

I agree with you, sir, that this controversy cannot be beneficial to our funds or to the reputation of the honorary staff, to whose devotion and untiring labours we are so much indebted, and can only regret that Colonel Garnham should thus seek to air his personal grievances—for the divisions at the various meetings show that he has but few supporters—regardless of any harm he may do to an institution doing such an exceptional amount of good work.

I am, etc., NORCLIFFE GILPIN,
Chairman, Committee of Management.

Chichester Infirmary, December 4th, 1906.

